# Supplementary material for: Rational Water and Nitrogen Regulation Can Improve Yield and Water–Nitrogen Productivity of the Maize (Zea mays L.)–Soybean (Glycine max L. Merr.) Strip Intercropping System in the China Hexi Oasis Irrigation Area
Source: Plants (Basel). 2025 Jul 4;14(13):2050. doi: 10.3390/plants14132050 (PMC12251900; doi:10.3390/plants14132050)
Supplement: Supplementary file 1 [file plants-14-02050-s001.zip › plants-3727613-supplementary.pdf]

Supplementary material

Figure. S1

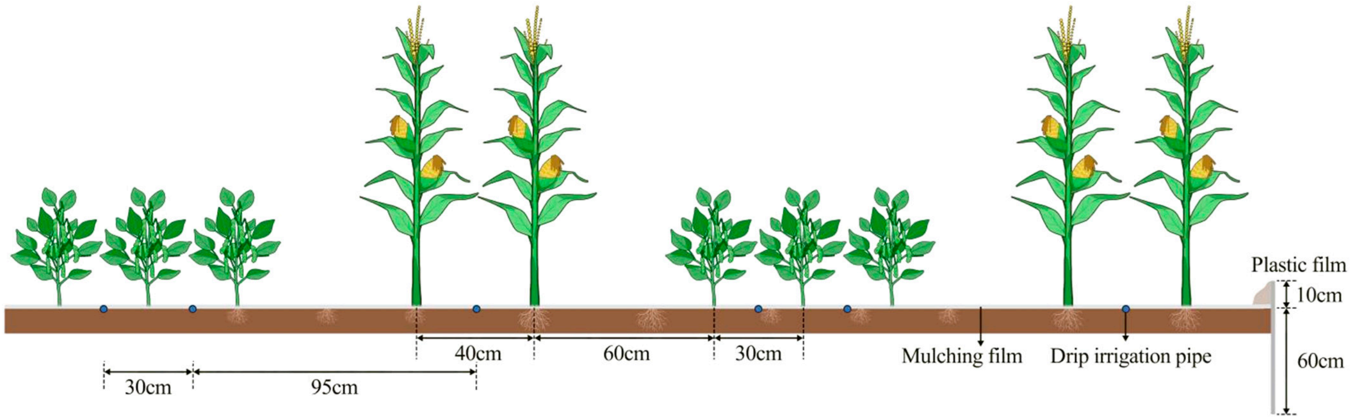

Figure S1. Field experiment of maize-soybean strip intercropping system.

Figure. S2

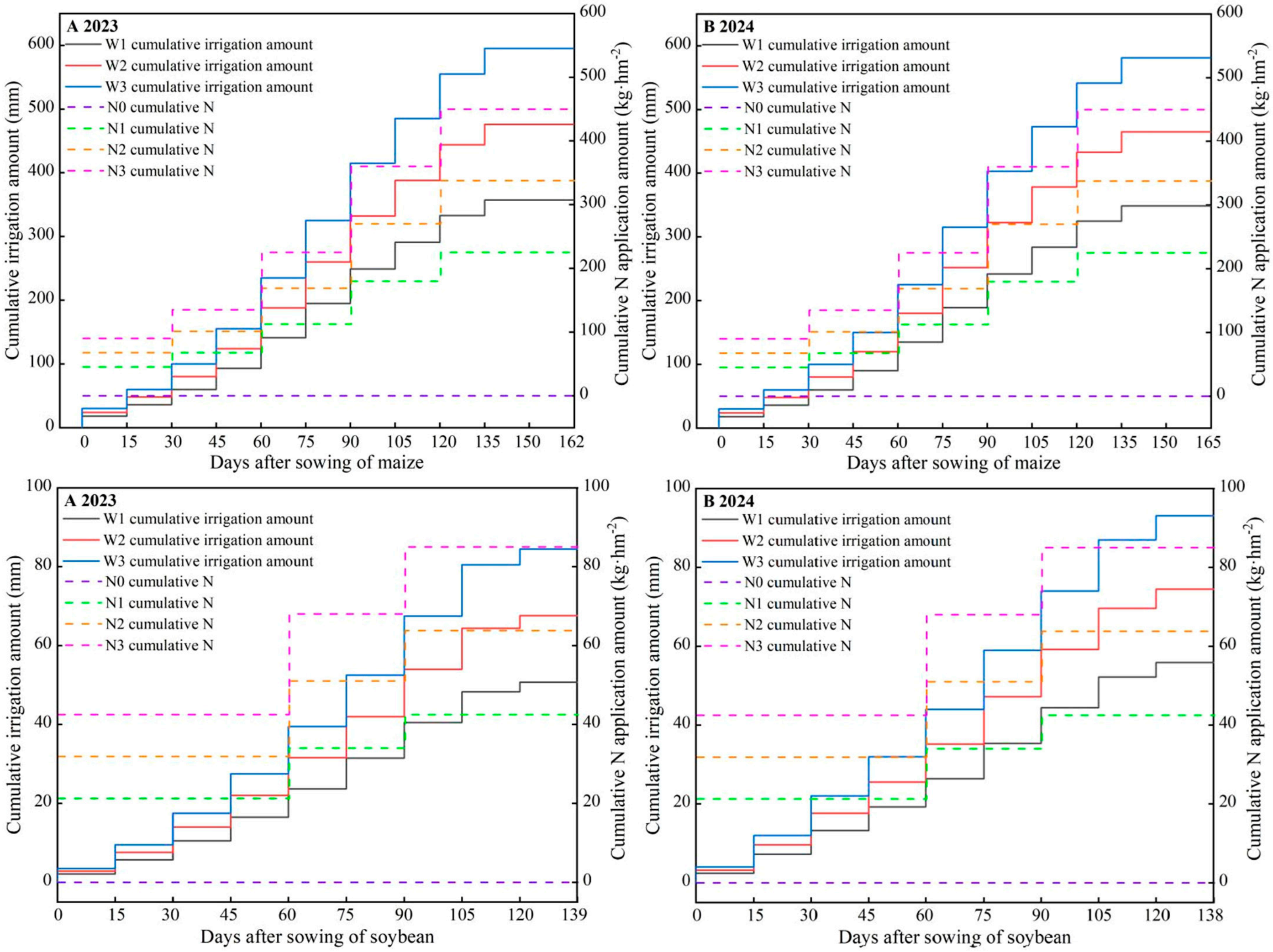

Figure S2. Design of different irrigation and fertilization systems of maize and soybean in strip intercropping system of 2023 and 2024.

Table S1

Table S1. Results of calculating weights based on the AHP hierarchical analysis method.

Hierarchy		Index	Local Weight	Final Weight	Consistency Test Parameter
Target Layer C	C_1	yield	0.5242	0.5242	C_R=0.0036＜0.1<br>λmax = 3.0037
	C_2	water-nitrogen productivity	0.2785	0.2785	
	C_3	quality	0.1973	0.1973	
Target Layer C_1	C_{11}	yield	0.3979	0.2086	C_R=0.0036＜0.1<br>λmax =3.0037
	C_{12}	ear weight	0.3524	0.1847	
	C_{13}	dry matter accumulation	0.2497	0.1309	
Target Layer C_2	C_{21}	water consumption	0.1134	0.0316	C_R=0.0070＜0.1<br>λmax =5.0312
	C_{22}	water productivity	0.2912	0.0811	
	C_{23}	irrigation water productivity	0.2279	0.0635	
	C_{24}	nitrogen partial factor	0.1805	0.0503	
	C_{25}	nitrogen agronomic use	0.1869	0.0521	
Target Layer C_3	C_{31}	crude fat	0.1468	0.0290	C_R=0.0136＜0.1<br>λmax =5.0607
	C_{32}	starch	0.2350	0.0464	
	C_{33}	crude protein	0.1620	0.0320	
	C_{34}	lysine	0.3300	0.0651	
	C_{35}	bulk density	0.1263	0.0249	

Table S2

Table S2. Weights of a single index determined by the entropy weight method.

Indices	C_{11}	C_{12}	C_{13}	C_{21}	C_{22}	C_{23}	C_{24}	C_{25}	C_{31}	C_{32}	C_{33}	C_{34}	C_{35}
Weights	0.078	0.066	0.081	0.092	0.085	0.060	0.072	0.093	0.087	0.065	0.087	0.059	0.076

Table S3

Table S3. The weight of a single index based on combination assignment.

Indices	C_{11}	C_{12}	C_{13}	C_{21}	C_{22}	C_{23}	C_{24}	C_{25}	C_{31}	C_{32}	C_{33}	C_{34}	C_{35}
Weights	0.195	0.172	0.126	0.038	0.081	0.063	0.053	0.056	0.035	0.048	0.038	0.065	0.030

Table S4

Table S4. Results of calculating weights based on the AHP hierarchical analysis method.

Hierarchy		Index	Local Weight	Final Weight	Consistency Test Parameter
Target Layer C	C_1	yield	0.4600	0.4600	C_R=0.0015＜0.1<br>λmax =3.0015
	C_2	water-nitrogen productivity	0.3189	0.3189	
	C_3	quality	0.2211	0.2211	
Target Layer C_1	C_{11}	yield	0.3750	0.1725	C_R=0.0000＜0.1<br>λmax =3.0000
	C_{12}	kernel weight per plant	0.3750	0.1725	
	C_{13}	dry matter accumulation	0.2500	0.1150	
Target Layer C_2	C_{21}	water consumption	0.1047	0.0334	C_R=0.0040＜0.1<br>λmax =5.0179
	C_{22}	water productivity	0.2571	0.0820	
	C_{23}	irrigation water productivity	0.2675	0.0853	
	C_{24}	nitrogen partial factor productivity	0.1853	0.0591	
	C_{25}	nitrogen agronomic use efficiency	0.1853	0.0591	
Target Layer C_3	C_{31}	crude protein	0.5000	0.1106	C_R=0.0000＜0.1<br>λmax =2.0000
	C_{32}	crude fat	0.5000	0.1106	

Table S5

Table S5. Weights of a single index determined by the entropy weight method.

Indices	C_{11}	C_{12}	C_{13}	C_{21}	C_{22}	C_{23}	C_{24}	C_{25}	C_{31}	C_{32}
Weights	0.092	0.084	0.105	0.106	0.059	0.086	0.102	0.105	0.140	0.122

Table S6

Table S6. The weight of a single index based on combination assignment.

Indices	C_{11}	C_{12}	C_{13}	C_{21}	C_{22}	C_{23}	C_{24}	C_{25}	C_{31}	C_{32}
Weights	0.154	0.152	0.113	0.050	0.077	0.086	0.069	0.069	0.117	0.113
